# Decision-making process of breastfeeding behavior in mothers with gestational diabetes mellitus based on health belief model

**DOI:** 10.1186/s12884-023-05527-3

**Published:** 2023-04-12

**Authors:** Pan Qian, Lixia Duan, Rujiao Lin, Xiwang Du, Dan Wang, Tieying Zeng, Chenxi Liu

**Affiliations:** 1 Nursing department in Tongji Hospital, Tongji Medical College, Huazhong University of Science and Technology, Wuhan, Hubei China; 2 School of Medicine and Health Management, Tongji Medical College, Huazhong University of Science and Technology, Wuhan, Hubei 430030, China

**Keywords:** GDM, Breastfeeding, HBM, Decision-making process

## Abstract

**Background:**

Gestational diabetes mellitus (GDM) threatens GDM mothers and their offspring’s health and breastfeeding is one of the most effective ways to decrease the risk. However, the prevalence of breastfeeding among GDM mothers is far from optimal and how GDM mothers develop their feeding behavior is still unclear. Thus, this study aimed to explore the formation of GDM mothers’ breastfeeding behaviors based on the health belief model (HBM).

**Methods:**

A questionnaire survey was conducted on 324 GDM mothers who have given birth within 6 months from January 1 to February 6, 2022. According to HBM, GDM mothers’ knowledge, the perceived threat from GDM, the perceived value of breastfeeding, self-efficacy, social support and GDM mothers’ breastfeeding behavior were measured. Exclusive breastfeeding (EBF) was defined as an infant who received only breast milk in the past 24 h before the survey. Structural equation modeling (SEM) was applied to explore how GDM mothers form their breastfeeding behaviors based on HBM.

**Results:**

The prevalence of EBF among GDM mothers was 33.95%. GDM mothers had limited knowledge of GDM (average 63.14% correct answer to 7 questions), especially poor on the long-term effect of GDM (39.81%) and protective effect of breastfeeding (34.57%-45.99%). Although GDM mothers showed high perceived benefits (Mean: 3.35, SD: 0.46), high self-efficacy (Mean: 3.43, SD: 0.97) and high level of social support for breastfeeding (Mean: 3.74, SD: 0.74), the various barriers (Mean: 2.20, SD: 0.47) hindered their success in EBF. The SEM results showed that a higher level of social support and more self-efficacy of breastfeeding resulted in a higher likelihood of EBF, while the higher level of knowledge of GDM, perceived higher barriers and benefits of breastfeeding and higher susceptibility to GDM consequences led to less EBF.

**Conclusion:**

To promote EBF, physicians’ education, emphasizing the protective effect of breastfeeding and how to correct breastfeeding, is highly recommended. In addition, social support for GDM mothers is also important to reduce their barriers to breastfeeding and help enhance self-efficacy in breastfeeding.

**Supplementary Information:**

The online version contains supplementary material available at 10.1186/s12884-023-05527-3.

## Introduction

As an important type of diabetes, Gestational Diabetes Mellitus (GDM) is a condition of glucose intolerance developing during pregnancy. According to International Diabetes Federation (IDF), 17% of the maternal population (aged 21–49 years old) suffered from GDM worldwide in 2021, affecting 21 million newborns [1]. The prevalence of the maternal population suffering from GDM during 2012–2015 in China was 17.5%, which showed an increasing trend in recent years [2].

Although GDM resolves after delivery for the majority of women, it is not uncommon for mothers and their offspring to suffer from multiple long-term health outcomes [3–6]. GDM mothers are more likely to progress to type II diabetes mellitus (T2DM) and cardiovascular disease (CAD) later in life, with over half of GDM mothers reported to develop T2DM after several years [4–6]. On the one hand, offspring exposure to GDM was related to a high risk of obesity and T2DM in their later life in addition to the high risk of macrosomia and neonatal hypoglycemia in the perinatal period [3, 7].

Breastfeeding has been demonstrated to be one of the most effective ways to improve the near-term and long-term prognosis of mothers with GDM and their offspring [8–10]. It is known that breastfeeding help delay or prevent GDM mothers from developing type II diabetes [8]. In addition, breastfeeding has been reported to be associated with a reduced risk of obesity and diabetes mellitus in GDM mothers’ offspring [9, 10]. Thus, GDM mothers are supposed to insist on breastfeeding to mitigate the health risks for themselves and their offspring.

However, the prevalence of breastfeeding among GDM mothers was far from optimal around the world, with 34.8% of GDM mothers exclusively breastfeeding (EBF) their infants which was far less than the target of 50% of the recommended criteria from the World Health Organization [11, 12]. The prevalence of exclusive breastfeeding (EBF) in infants under 6 months was 29.2% according to a national survey in China and the prevalence was lower in GDM mothers [13].

However, GDM mothers’ formation and decision-making process regarding their feeding behavior was still unclear. Existing studies concentrated on healthy mothers and found that pathological factors [14], psychological factors [13], knowledge of breastfeeding [15], perceived barriers to breastfeeding [16], self-efficacy [17], social support [16] and personal characteristics [12, 15] was associated with maternal breastfeeding intention and actual behavior. However, this is not the situation for GDM mothers [18–20]. It was shown that GDM mothers were at greater risk of delayed lactation initiation [21] and higher risk of adverse outcomes from GDM (such as neonatal hypoglycemia, macrosomia and cesarean section) [22], which put more burden and barriers compared with healthy mothers. Studies also found that mothers with GDM required more social support from professionals and their families and more self-efficacy in breastfeeding to insist on breastfeeding compared with mothers without GDM [16, 23]. Though a few studies tried to explore potential factors related to breastfeeding in GDM mothers, they failed to link them with GDM mothers’ actual feeding behaviors. Instead, feeding intention was treated as proxy indicator [17, 24]. There is also a shortage of research documenting how GDM mothers decide to breastfeed in China, and the decision-making process of their feeding behavior is still unclear.

Therefore, it is necessary to understand the decision-making process and key factors of their feeding behaviors. The current study investigated GDM mothers’ breastfeeding behaviors and potential determinants based on the health belief model (HBM).

## Participants and Methods

### Settings

This study was conducted in the Obstetrics Department of Tongji Hospital, Tongji Medical College, Huazhong University of Science and Technology (Wuhan, China). Tongji hospital is one of the largest tertiary general hospitals in central China and serves patients with acute and critical illnesses from all over Hubei Province and surrounding provinces with more than 6000 beds. As the designated maternal critical care center by governments, the obstetrical department of Tongji Hospital has a total of 6 wards with over 200 beds. In 2021, Tongji Hospital serves approximately 6,800 pregnant women’s delivery, of whom 18% are diagnosed with GDM.

### Theoretical framework

**Fig. 1:**
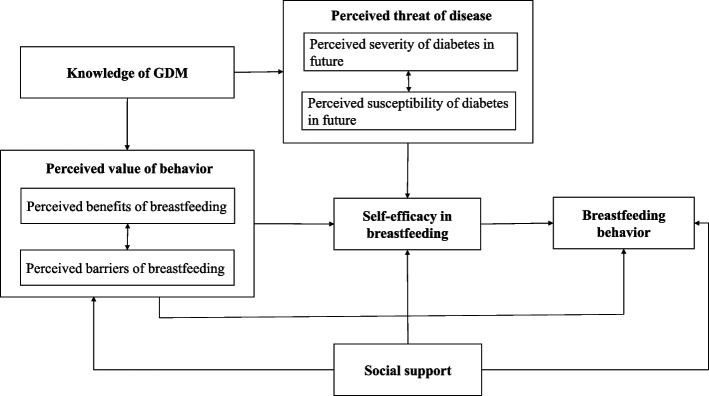
Theoretical framework

Health Belief Model (HBM) was adopted to guide the study design in the current study. Based on HBM, person’s perceived threat of an illness together with a person’s belief in the effectiveness of the recommended health behavior will predict the likelihood the person will adopt the behavior [25]. We modified the model based on our previous qualitative interviews (published elsewhere [26]). The model consists of 8 subdimensions:1) Knowledge of GDM; 2) Perceived susceptibility of GDM; 3) Perceived severity of GDM; 4) Perceived benefits of breastfeeding; 5) Perceived barriers of breastfeeding; 6) Self-efficacy in breastfeeding; 7) Social support; 8) Feeding behavior (Fig. 1).

Based on the HBM model (Fig. 1), we hypothesized that knowledge of GDM can shape the perceived threat of GDM and the perceived value of breastfeeding. The perceived value of breastfeeding can directly link to actual breastfeeding behavior. Meanwhile, the impact of the perceived value of breastfeeding or perceived threat of disease on actual behavior can be mediated by self-efficacy in breastfeeding. Furthermore, social support can directly influence breastfeeding behavior and, at the same time, indirectly affect behavior by reducing barriers to breastfeeding.

### Survey instruments

A 60-item questionnaire was developed measuring the above 8 sub-dimensions and personal characteristics of participants associated with breastfeeding (Supplementary S1).

Knowledge was measured using 7 questions based on the previous literature [24]. Five of these questions were asking the respondents to make a judgement on the adverse outcome of GDM (obesity, type 2 diabetes, etc.), and two questions addressed the effect of breastfeeding on reducing long-term adverse outcomes in women with GDM and their newborns. Each question contained three choices (true, false, I don’t know), and only one answer was correct.

The GDM-related health belief, including the perceived threat of GDM and perceived value of breastfeeding, was measured using 27 items along a four-point Likert scale, with each subscale containing a minimum of four items. These items were developed and revised based on our qualitative interviews [26] and previous studies applying Health Belief Model [27]. The perceived threat of GDM was measured via perceived susceptibility and severity of GDM. Four items concerning perceived susceptibility were asking respondents about their or their newborns’ possibility of adverse of GDM, and six items on perceived severity of GDM were investigating respondents’ subjective judgement on the harm of GDM to their physical life and the economic burden. In addition, the perceived value of breastfeeding was determined by both perceived benefits (9 items) and barriers (8 items) of breastfeeding, respectively.

Social support for breastfeeding was measured using 12 items along a five-point Likert scale, which was adapted from the Berlin Social Support Scale and revised in the context of breastfeeding [28]. Self-efficacy in breastfeeding was measured using the Breastfeeding Self-Efficacy Scale-Short Form (BSES-SF) with 14 items [29].

Breastfeeding behavior was measured using a 24-h reported food recall method asking respondents if a mother had given her newborn breast milk or other substitutes (Formula milk, goat’s milk, sugar water).

Personal characteristics included respondents’ age, education, marriage, mode of delivery, parity, neonatal month age, residence, genetic history of diabetes, work status, drinking history and smoking history.

The questionnaire was piloted on 15 pregnant women with GDM to evaluate the reliability and validity. According to their feedback, items were revised or removed. The reliability and validity of the final questionnaire were confirmed by confirmatory factor analysis (CFA) and Cronbach’s alpha. Acceptable Cronbach’s alpha was identified for all sub-dimension of the instrument (α = 0.763–0.951). The confirmatory factor analysis showed that the model fit index of the overall model was good with Root Mean Square Error of Approximation (RMSEA) = 0.040(< 0.080), Comparative Fit Index (CFI) = 0.980 (> 0.950), Tucker-Lewis Index (TLI) = 0.978 (> 0.950).

### Sampling and data collection

All pregnant women diagnosed with GDM who give birth within six months in Tongji Hospital, Wuhan, Hubei Province from April 2021 to December 2021 were included in the current study. GDM mothers who had severe complications and contraindications to breastfeeding were excluded.

The GDM diagnosis was based on the GDM clinical guideline issued by the National Health Commission of China [30]. A 75 g oral glucose tolerance test (OGTT) was performed between 24 and 28 weeks of gestation. Gestational diabetes mellitus (GDM) is diagnosed if the fasting blood glucose was greater than 0.51 mmol/L or 10.0 mmol/L 1 h after taking glucose, or if the blood glucose value was greater than 8.5 mmol/L 2 h after taking glucose.

A web-based survey was distributed through the Wenjuanxing platform over the period from January 1 to February 6, 2022. Written informed consent from each respondent was obtained. The survey took 10–15 min on average and respondents were encouraged to consult the researcher if they feel unclear about the questionnaire. A token gift (roughly $2) was given to the participants after the completion of the survey.

A total of 1004 pregnant women were approached. Among them, 885 women met the inclusion criteria and were invited to participate. 401 respondents agreed and completed the survey with a response rate of 45.3%. Finally, 324 respondents were included after removing the infants older than 6 months. Selective bias was not indicated since no significant differences were identified in the demographic characteristic between GDM mothers who participated and who did not (Supplementary S2).

### Ethical considerations

Ethics approval has been provided for our study by the Tongji hospital ethics committee. The ethic number is NO.TJ-IRB20210755.

### Data analysis

According to the WHO definition of exclusive breastfeeding, breastfeeding behavior in this study was defined as an infant who received only breast milk [31]. The infant feeding area graphs were constructed.

The responses to knowledge items were coded as a binary variable (1 = correct answer, 0 = wrong answer/I don’t know). For each respondent, knowledge of GDM was assessed based on the total number of correct answers (ranging from 0 to 7).

Each GDM-related health belief was coded along a four-point scale, with a higher score indicating more agreement with the perceived threat of disease and perceived value of breastfeeding. The average scores for each sub-dimension were calculated (ranging from 1 to 4).

Social support and self-efficacy were coded along a five-point scale, with a higher score indicating more support from family members, friends and professionals or greater willingness and ability to breastfeed, respectively. The average scores were calculated (ranging from 1 to 5).

A structural equation modeling (SEM) was applied to explore the decision-making process of breastfeeding behavior among GDM mothers based on HBM (Fig. 1). Model estimation was based on the weighted least squares with adjusted mean and variance (WLSMV). Several indexes were used to evaluate the goodness-of-fit of the proposed SEM model, including REMSA < 0.080, CFI > 0.950, TLI > 0.950, WRMR < 1.

Stata 14.0 and Mplus 7.0 were used for statistical analysis. A *p*-value < 0.05 was considered statistically significant.

## Results

### Characteristics of respondents

A total of 324 mothers with GDM participated in the current study (Table 1). The majority (94.44%) of the respondents were between 25 and 39 years old and more than half of newborns were less than 4 months (57.72%). As for the mode of delivery, more than half of the births were by caesarean Sect. (62.65%). Over three-fifths (66.36%) of respondents were first birth. After childbirth, women mainly lived with their husbands (37.35%) or with their husband and parents (50.31%). A few respondents have a history of smoking (3.09%) and drinking (10.80%). About one-third (31.48%) of respondents admitted a family history of diabetes.

**Table 1:** Characteristics of respondents

Characteristics	Mean ± SD/N (%)
Age (years)	
< 25	9 (2.78)
25–30	96(29.63)
30–35	140 (43.21)
35–40	70 (21.60)
40–45	9 (2.78)
Level of education	
High school or technical secondary school and below	56 (17.28)
Junior college	86 (26.54)
Undergraduate	140 (43.21)
Master or above	42 (12.96)
Living structure	
Living alone	11 (3.40)
Living with husband	121 (37.35)
Living with parents	27 (8.33)
Living with husband and parents	163 (50.31)
Others	2 (0.62)
Living place	
Urban	304 (93.83)
Rural	20 (6.17)
Working status	
Yes	59 (18.21)
No	265 (81.79)
Mode of delivery	
Vaginal delivery	121 (37.35)
Caesarean section	203(62.65)
Parity	
First birth	215 (66.36)
Second birth	103 (31.79)
Third birth or above	6 (1.85)
Age of neonates (month)	
0–3	187 (57.72)
4–6	137 (42.28)
Family history of diabetes	
Yes	102 (31.48)
No	222 (68.52)
Drinking history	
Yes	35 (10.80)
No	289 (89.20)
Smoking history	
Yes	10 (3.09)
No	314 (96.91)

### Breastfeeding behavior

**Fig. 2:**
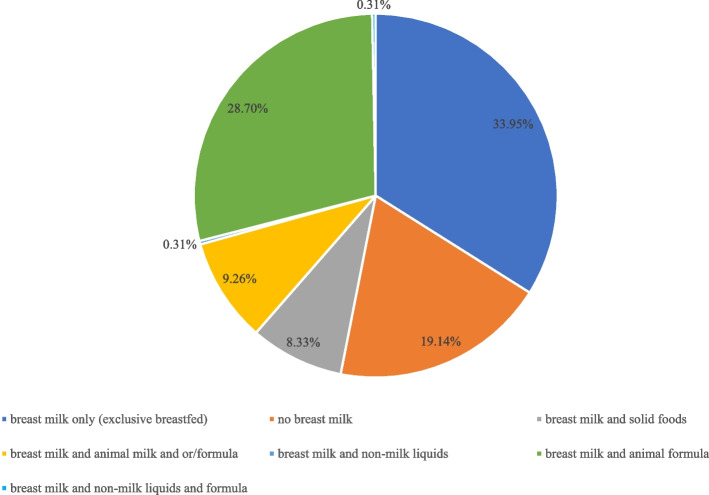
Feeding practice in the mothers with GDM

The prevalence of exclusive breastfeeding was 33.95% in the mothers with GDM, followed by the proportion 28.70% of mothers feeding their child with breast and animal formula. Nearly one-fifth of mothers did not breastfeed their child within 24 h before the survey. For more details on breastfeeding practice see Fig. 2.

### Determinants of feeding behaviors

On average, the respondents answered over four questions correctly (SD = 1.80) of a total of 7 (Table 2). Mothers had a high awareness (92.28%) of the possible risk of macrosomia and obstructed labor due to GDM. Maternal knowledge of their own risk of long-term diabetes, postnatal hypoglycemic symptoms in the newborn, and common complications of gestational diabetes were relatively good, with a correct rate of 70%-90%. However, participants showed limited knowledge about the future risk of GDM to the newborn, as well as the important role of breastfeeding in reducing the future risk of GDM to mothers and newborns (correct answer rate < 50%).

**Table 2:** Respondents’ knowledge regarding GDM

Items	Correct answer n (%)
GDM pregnancy is more likely to have pregnancy-induced hypertension. (T)	217 (66.98)
GDM pregnancy is a high risk for dystocia due to gigantic childbirth. (T)	299 (92.28)
Newborns in GDM may develop hypoglycemia within 1 to 3 h after delivery. (T)	241 (74.38)
A woman with GDM is more likely to have type 2 diabetes, obesity, and cardiovascular disease after delivery than normal pregnancy. (T)	285 (87.96)
Newborns in GDM have a similar likelihood to develop type 2 diabetes, obesity, and cardiovascular disease as normal newborns. (F)	129 (39.81)
Breastfeeding can reduce the likelihood of type 2 diabetes, obesity, and cardiovascular disease in women with GDM. (T)	149 (45.99)
Breastfeeding does not reduce the likelihood of type 2 diabetes, obesity, and cardiovascular disease in newborns of GDM. (F)	112 (34.57)
Overall scores (Mean ± SD)	4.42 ± 1.80

In terms of the future threat from GDM, respondents generally held a neutral attitude towards the perceived susceptibility to the long-term harm of GDM (2.53 ± 0.52) (Table 3). Though GDM mothers generally believed that they had a higher risk of diabetes and obesity in the future, they showed a negative attitude toward their newborns’ risk (Supplementary S3).

**Table 3:** Respondents’ determinants of breastfeeding based on HBM

Constructs	Number of items	Range	Mean	SD	Cronbach’s alpha
Perceived susceptibility	4	1–4	2.53	0.52	0.7754
Perceived severity	6	1–4	2.96	0.57	0.8717
Perceived benefits	9	1–4	3.35	0.46	0.8801
Perceived barriers	8	1–4	2.20	0.47	0.7639
Social support	12	1–5	3.74	0.74	0.9451
Self-efficacy	14	1–5	3.43	0.97	0.9508

On the other hand, they perceived the severity of long-term harm of GDM (2.96 ± 0.57) (Table 3), believing that developing diabetes in the future could have serious economic and health consequences for themselves and their newborns (Supplementary S3).

Regarding the perceived value of breastfeeding, respondents commonly reported perceived the benefits of breastfeeding (3.35 ± 0.46) (Table 3), agreeing that breastfeeding is nutritious and safe and can enhance the parent–child bond and improve the child’s immunity (Mean score > 3.24, SD: 0.49–0.73). However, maternal awareness of the benefits of breastfeeding to reduce the future diabetes of themselves (3.06 ± 0.75) and their child (3.04 ± 0.76) was insufficient (Supplementary S3). On the other hand, respondents reported a high level of perceived barriers to breastfeeding (2.20 ± 0.47). Insufficient breast milk supply, believing that formula milk could provide the same nutrition as breast milk, and the impact of breastfeeding on daily life were considered important barriers to breastfeeding (Mean score > 2.3, SD: 0.78–0.93). In contrast, neonatal jaundice and breast distension engorgement were not major obstacles to breastfeeding (Mean score < 2.3, SD: 0.63–0.67).

Generally, respondents showed a high level of self-efficacy in breastfeeding (3.43 ± 0.97) (Table 3) and received a high level of social support from families, friends and society (3.74 ± 0.74). The details of responses could be found in supplementary S3.

### Formation of breastfeeding behavior based on HBM

**Fig. 3:**
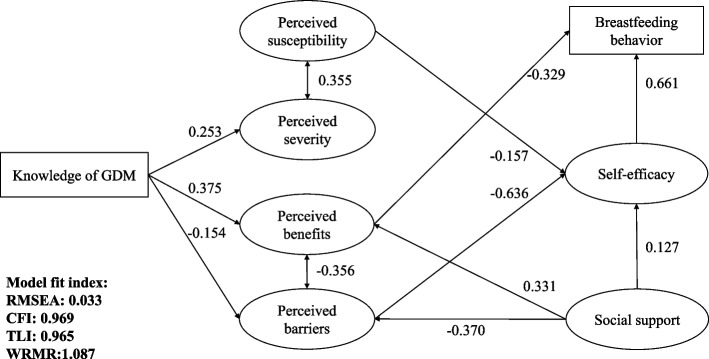
Formation of feeding behaviors of GDM mothers based on HBM. Legends: Results based on SEM and only significant pathways (*p* < 0.05) were reported with standardized path coefficients

The SEM confirmed the theoretical framework for the breastfeeding behavior of GDM based on HBM (Fig. 3) with an excellent model fit index (RMESA = 0.033, CFI = 0.969, TLI = 0.965, WRME≈1 (1.087)).

Higher knowledge scores were found to be linked with fewer perceived barriers (β = -0.154, *p* = 0.034) and more perceived benefits (β = 0.375, *p* < 0.0001) of breastfeeding. It was also associated with a higher level of perceived severity (β = 0.253, *p* = 0.010), but not significantly related to higher perceived susceptibility (*p* = 0.697) of GDM long-term consequences. Moreover, a higher level of social support was associated with higher perceived benefits (β = 0.331, *p* < 0.0001) and fewer perceived barriers (β = -0.370, *p* < 0.0001).

Furthermore, higher perceived barriers (β = -0.636, *p *< 0.0001), higher perceived susceptibility (β = -0.157, *p* = 0.005) and less level of social support (β = 0.127, *p* = 0.006) were associated with GDM mothers’ less self-efficacy of breastfeeding. Respondents who showed higher self-efficacy (β = 0.661, *p* < 0.0001) and perceived less benefit of breastfeeding (β = -0.329, *p* < 0.0001) were more likely to breastfeed the newborns.

To explore whether the relationship of breastfeeding behavior based on HBM varied between subgroups of mothers. Respondents’ characteristics (such as age, mode of delivery, and history of smoking) were further included as covariates. Age, genetic history of diabetes, drinking history, education level, parity, working status, age of neonatal, and occupation were found to be significantly associated with GDM mothers’ perceived health belief towards breastfeeding. (Details see Supplementary S4).

In sum, knowledge of GDM had indirect effects on breastfeeding via changing mothers’ perceived benefits and barriers of breastfeeding and the total effect is negative (total effect: -0.030). GDM mothers’ perceived barriers to breastfeeding (total effect: -0.303), perceived susceptibility of GDM long-term consequences (total effect: -0.104), and reception of social support (total effect: 0.137) were able to influence their breastfeeding behaviors via changing self-efficacy. However, only an increase in social support can result in a higher likelihood of GDM mothers’ breastfeeding. Finally, mothers’ self-efficacy (total effect: 0.596) and perceived benefits of breastfeeding (total effect: -0.372) directly influenced their feeding behaviors.

## Discussion

### Main findings

The study adopted HBM to explore how GDM mothers form their feeding behaviors as well as its potential determinants. It seemed that GDM mothers showed a relatively low rate of breastfeeding and they commonly did not recognize the long-term effect of GDM and the protective effect of breastfeeding on themselves and their offspring. Although GDM mothers showed high self-efficacy and a high level of social support for breastfeeding, the various barriers hindered their success in the such feeding patterns. Based on the well-fitting SEM results, it was shown that knowledge of GDM can shape one’s perceived benefits and barriers of EBF and the perceived severity of GDM. Perceived barriers to breastfeeding and perceived susceptibility and social support may alter feeding behaviors by changing GDM mothers’ self-efficacy. Perceived benefits and self-efficacy of breastfeeding were identified as two main significant predictors of EBF.

### Comparison to existing studies

#### Breastfeeding behavior

Despite the well-known importance of breastfeeding for mothers with GDM and their offspring, few studies had reported the rate of EBF among GDM mothers in China. The prevalence of EBF in this study among mothers with GDM was 33.95%, which was consistent with results from western China, in which a prevalence of EBF of 36.9% in mothers with GDM was identified (at 6 months postpartum) [23]. However, it was lower than the global prevalence of 45.7% under 6 months among low- and middle-income countries [32] and far less than the national target of 50% issued by WHO and the National Health Commission of the People’s Republic of China [33].

#### Knowledge of GDM, GDM-related health beliefs, social support, and self-efficacy toward breastfeeding

The respondents scored 63.14% on average in correct answers about gestational diabetes mellitus (4.49 out of 7 questions). This result revealed a low level of GDM knowledge in the study participants., compared with 66.6%-89.3% of pregnant women without GDM who have an average or good knowledge of GDM in India [34–37]. Studies from Korea [24], Malaysia [38], and Bangladesh [17] also reported a low level of knowledge of GDM with a prevalence of 30.9%-54.9%. Specifically, the knowledge gap on the long-term consequences of GDM on offspring and the protective effect of breastfeeding on GDM mothers and their offspring was as large as in other regions [17, 24, 39], indicating there is an urgent need to improve the awareness of GDM-related knowledge.

In terms of the perceived threat of gestational diabetes, mothers tend to underestimate the long-term threat of gestational diabetes to themselves and their newborns, which was reflected in the negative attitude towards their newborns’ risk of long-term diabetes and potential economic and health consequences for themselves and their newborns. The result was consistent with the previous studies [40, 41]. Considering the fact that GDM resolves after delivery for the majority of women, GDM mothers mistakenly believed that GDM is merely related to pregnancy to underestimate the long-term consequences of GDM [26]. On the other hand, GDM patients formed their health beliefs and behaviors based on their contact with health professionals [42] and other patients [43]. Health professionals’ neglectful attitudes and practices may shape the patients’ regardless attitudes and practices toward GDM. This phenomenon is more likely to occur under the circumstance of excessive workload since physicians have insufficient time to interpret the relevant knowledge [26].

Although respondents showed a generally positive attitude towards breastfeeding, maternal awareness of the benefits of breastfeeding to reduce the future diabetes of themselves and their child was insufficient. The low awareness of the protective effect of breastfeeding may hinder the promotion of breastfeeding behavior among GDM mothers. In terms of perceived barriers, insufficient breast milk, limited time and energy, and the impact of breastfeeding on daily life were reported to be important barriers to breastfeeding in this study, which is similar to a report issued by the Chinese Development Research Foundation [44]. The result was also consistent with a study from Australia showed that insufficient milk, breastfeeding problems and return to work were the main reasons for early cessation of breastfeeding at ≤ 3 months [45]. However, there was no difference between mothers with GDM and healthy mothers in the prevalence of reporting insufficient milk [46] in the U.S.A. In contrast, GDM mothers seemed more likely to report that their families and physicians preferred formula feeding and their offspring had limited interest in breastfeeding [47]. In addition, GDM mothers also showed high self-efficacy in breastfeeding and received a high level of social support from families, friends and society, which is consistent with the results from the previous study in China [48, 49] and Turkey [50].

#### Decision-making process of breastfeeding behavior

In terms of the formation of feeding behaviors, this study confirmed that self-efficacy in breastfeeding was one significant predictor of EBF among mothers with GDM, which was consistent with the result of Brockway’s systematic review that improving breastfeeding self-efficacy was significantly associated with breastfeeding [51]. In contrast, previous studies showed that perceived benefits were reported to be a significant factor in better breastfeeding intention and actual behavior [17, 24]. However, the current study showed a contradictory result. One plausible reason is reporting bias from respondents. The perceived benefits of breastfeeding in the current study may measure expected rather than actual attitudes towards breastfeeding of GDM mothers. Considering the widespread advocacy of breastfeeding in society and the high level of social support from family, GDM mothers may be under high pressure to indicate the high benefit of breastfeeding, while the actual breastfeeding behavior is less adopted due to more perceived barriers. A previous study has shown that GDM mothers commonly perceived a high level of advocacy of breastfeeding from family and society, while the final breastfeeding behaviors were mainly determined by GDM mothers [26]. However, such an effect warranted further study.

Congruent with previous studies [49, 52–56], although social support was not directly associated with breastfeeding behavior, it could improve EBF by enhancing self-efficacy in breastfeeding. One study conducted in the Northeast United States also showed that support from the network did not directly affect breastfeeding duration and pattern, but it did have an indirect influence through self-efficacy [56]. This suggests that enhancing a support network for breastfeeding is one important way to promote breastfeeding self-efficacy and help form EBF for GDM mothers.

Knowledge of GDM also shaped people's perceptions of disease and health behaviors. This study showed that mothers with more knowledge of GDM perceived more benefits and fewer barriers to breastfeeding and more severity of adverse consequences of GDM. Previous studies have illustrated that increased awareness of breastfeeding’s benefits [57] and more positive attitudes [58, 59] towards breastfeeding and fewer breastfeeding difficulties [58] were associated with better breastfeeding practice. Therefore, it is feasible to promote breastfeeding behavior in mothers with GDM by improving their knowledge.

### Implication for clinical practices and policy

Based on the current study, the intervention for promoting EBF should be multi-faceted with the participation of the HCPs, GDM mothers and their social support network.

Firstly, healthcare providers’ education on GDM and EBF according to clinical guidelines should be implemented to ensure GDM mothers form a correct understanding of disease and health behaviors. Based on our findings, education should emphasize the protective effect of breastfeeding on adverse outcomes of GDM. Considering the fact that HCPs served as the authority to form the public’s health beliefs, doctors, nurses and midwives all may play an important role.

Secondly, improving GDM mothers’ self-efficacy in breastfeeding is critical and education about breastfeeding knowledge and skills in the whole process from prenatal to postpartum was also recommended. Studies from the U.S.A [60] and China [23] have illustrated that breastfeeding education via class and text message throughout prenatal and postnatal stages significantly increases GDM mothers’ self-efficacy, resulting in a higher likelihood of them adopting breastfeeding and prolonging their breastfeeding duration.

Finally, social support is warranted to reduce barriers to breastfeeding and help enhance GDM mothers’ self-efficacy in breastfeeding. Studies have shown that support from health personnel, mass media, family members, and friends was the most important factor in the persistence of breastfeeding [55, 56].

### Strength and limitations

To the best of our knowledge, this is the first study to investigate the breastfeeding behavior among mothers with GDM based on HBM, which linked the actual breastfeeding behavior of GDM mothers with potential determinants. The such design enabled us to comprehensively understand the decision-making process of breastfeeding behavior among mothers with GDM and the findings have significant implications for policy development and intervention design in the context of developing countries.

There are also some limitations in the study. The participants in our study were recruited from a tertiary hospital in the center of a city (Wuhan) in China and the majority of them lived in the urban area and had received a high school education or above. Furthermore, the cesarean rate was very high in the study population. Attempts to generalize the finding of this study to other conditions should be cautious. Future studies are needed to include a more representative sample to confirm the results. In addition, more research is needed to determine to what extent these results reflect their actual behavior due to the GDM mothers’ self-reported responses.

## Conclusion

Mothers with GDM showed a low rate of breastfeeding and they commonly did not recognize the long-term effect of GDM and the protective effect of breastfeeding on themselves and their offspring. Although GDM mothers showed high self-efficacy and a high level of social support for breastfeeding, the various barriers hindered their success in the such feeding pattern. During the formation of feeding behaviors, GDM mothers’ self-efficacy, perceived benefits and barriers of breastfeeding, perceived severity of GDM long-term consequence, knowledge of GDM and social support played roles. To improve EBF for GDM mothers, physicians’ education, emphasizing the protecting effect of breastfeeding and how to correct breastfeeding, is highly recommended. In addition, social support for GDM mothers is also important to reduce their barriers to breastfeeding and help enhance self-efficacy in breastfeeding.

## Supplementary Information

**Additional file 1: Supplementary File 1.** Questionnaire on breastfeeding behavior and its determinants in patients with gestational diabetes mellitus. **Table S1-1.** **Supplementary File 2.** Analysis of demographic characteristics between 324 respondents and 885 pregnant women with gestational diabetes mellitus. **Supplementary File 3.** The Strengthening the Reporting of Observational Studies in Epidemiology (STROBE) checklist. **Supplementary File 4.** Respondents’ determinants of breastfeeding based on HBM. **Table S4-1.** Perceived susceptibility.** Table S4-2.** Perceived severity. **Table S4-3.** Perceived barrier. **Table S4-4.** Perceived benefit. **Table S4-5.** Social support. **Table S4-6.** Self-efficacy. **Supplementary File 5.** Results of covariates on the breastfeeding behavior based on HBM.

## Data Availability

The datasets used and analyzed during the current study are available from the corresponding author on reasonable requests.

## Abbreviations

GDM: Gestational Diabetes Mellitus
HBM: Health Belief Model
EBF: Exclusive Breastfeeding
IDF: International Diabetes Federation
CAD: Cardiovascular Disease
BSES-SF: Breastfeeding Self-Efficacy Scale-Short Form
CFA: Confirmatory Factor Analysis
CFI: Comparative Fit Index
TLI: Tucker-Lewis Index
WRMR: Weighted Root Mean Square Residual
OGTT: Oral Glucose Tolerance Test
WHO: World Health Organization
