# Supplementary material for: Decision-making process of breastfeeding behavior in mothers with gestational diabetes mellitus based on health belief model
Source: BMC Pregnancy Childbirth. 2023 Apr 12;23:242. doi: 10.1186/s12884-023-05527-3 (PMC10091643; doi:10.1186/s12884-023-05527-3)
Supplement: Supplementary file 1 — Additional file 1: Supplementary File 1. Questionnaire on breastfeeding behavior and its determinants in patients with gestational diabetes mellitus. Table S1-1. Supplementary File 2. Analysis of demographic characteristics between 324 respondents and 885 pregnant women with gestational diabetes mellitus. Supplementary File 3. The Strengthening the Reporting of Observational Studies in Epidemiology (STROBE) checklist. Supplementary File 4. Respondents’ determinants of breastfeeding based on HBM. Table S4-1. Perceived susceptibility. Table S4-2. Perceived severity. Table S4-3. Perceived barrier. Table S4-4. Perceived benefit. Table S4-5. Social support. Table S4-6. Self-efficacy. Supplementary File 5. Results of covariates on the breastfeeding behavior based on HBM. [file 12884_2023_5527_MOESM1_ESM.docx]

- **Supplementary File 1 Questionnaire on breastfeeding behavior and its determinants in patients with gestational diabetes mellitus**

**Table S1-1**

| **Measurement (Choices)** | **Items** |
| --- | --- |
| **Knowledge of GDM**  **(Yes/ No/ Unclear)** | 1. GDM pregnancy is more likely to have pregnancy induced hypertension. (T)  2. GDM pregnancy is a high risk for dystocia due to gigantic childbirth. (T)  3. Newborns in GDM may develop hypoglycemia within 1 to 3 hours after delivery. (T)  4. A woman with GDM is more likely to have type 2 diabetes, obesity, and cardiovascular disease after delivery than normal pregnancy. (T)  5. Newborns in GDM have similar likelihood to develop type 2 diabetes, obesity, and cardiovascular disease as normal newborns. (F)  6. Breastfeeding can reduce the likelihood of type 2 diabetes, obesity, and cardiovascular disease in women with GDM. (T)  7. Breastfeeding does not reduce the likelihood of type 2 diabetes, obesity, and cardiovascular disease in newborns of GDM. (F) |
| **Perceived susceptibility**  **(absolutely impossible/ impossible/ possible/ very possible)** | 1. How likely do you think you are to develop chronic diabetes in the future?  2. How likely do you think it is that your child will develop chronic diabetes in the future?  3. How likely do you think it is that your child will be obese as an adult?  4. How likely do you think you are to become obese in the future? |
| **Perceived severity**  **(not at all/ not serious/ serious/ very serious)** | 1. If you develop chronic diabetes in the future, do you think its consequences will be serious?  2. If you develop chronic diabetes in the future, how likely do you think you are to have serious adverse consequences of your diabetes (diabetic foot, cataracts, etc.)?  3. How likely are you to experience severe financial burden if you develop chronic diabetes in the future?  4. If your child develops chronic diabetes in the future, do you think the consequences will be serious?  5. If your child develops chronic diabetes in the future, how likely do you think it is that your child will have serious adverse consequences of diabetes (diabetic foot, cataract, etc.)?  6. If your child develops chronic diabetes in the future, how likely do you think it is that your child will have a serious financial burden from diabetes? |
| **Perceived barriers**  **(strongly agree/ agree/ disagree/ strongly disagree)** | 1. I don't have enough breast milk to allow me to breastfeed exclusively.  2. Breastfeeding affects my body image.  3. I don't have enough time and energy to breastfeed.  4. breast swelling would make me consider giving up breastfeeding.  5. Breastfeeding can make jaundice last longer in babies.  6. it is difficult for me to breastfeed.  7. Breastfeeding interferes with my daily life and rest.  8. I think formula can give babies exactly the same nutrition. |
| **Perceived benefits**  **(strongly agree/ agree/ disagree/ strongly disagree)** | 1. Breastfeeding is economical.  2. breastfeeding is convenient.  3. breast milk strengthens the child's resistance.  4. breastmilk is rich in nutrients.  5. breast milk is natural and safe.  6. breastfeeding can increase parent-child bonding.  7. Breastfeeding helps me recover faster from childbirth.  8. Breastfeeding reduces the likelihood of my developing diabetes in the future  9. Breastfeeding can reduce the likelihood of my child developing diabetes in the future |
| **Self-efficacy in breastfeeding**  **(Never/ seldom/ sometimes/ often/always)** | 1. I can always be sure that my baby is getting enough breast milk.  2. I have always managed to cope successfully with the challenges of breastfeeding.  3. I was always able to breastfeed exclusively without formula.  4. I always make sure my baby can hold the nipple well during feeding.  5. I always manage breastfeeding situations in a way that I am satisfied with.  6. I was always able to finish breastfeeding even when my child cried.  7. I was always able to breastfeed willingly from the beginning to the end.  8. I am always comfortable breastfeeding even when my family is around.  9. I am always satisfied with my breastfeeding experience.  10. I have always been able to handle the fact that breastfeeding is time consuming.  11. I can always get my child to empty one breast before sucking the other.  12. I can always use breast milk every time I feed my child.  13. I always find ways to keep up with my child's feeding needs.  14. I always know when my child is finished breastfeeding. |
| **Social support**  **(strongly agree/ agree/ neutral/ disagree/ strongly disagree)** | 1. During the feeding of my child, someone was always there for me when I needed help.  2. I always have someone around me to share my joys and sorrows in feeding my children.  3. My family was always trying to help me in the feeding of my child.  4. I received the emotional help and support I needed from my family during the feeding of my child.  5. I have people around me who make me feel really comfortable during the feeding of my child.  6. My friends always try to help me in the feeding process of my children.  7. I can rely on my friends if I encounter difficulties in feeding my child.  8. I can talk to my family about the difficulties I face during the feeding of my child.  9. I have friends who can share my joys and sorrows in feeding my child.  10. having people in my life who care about my feelings during the feeding of my child.  11. my family is willing to help me make decisions when it comes to feeding my child.  12. I can discuss the difficulties I face in feeding my child with my friend. |
| **Breastfeeding behavior**  **(Yes/ No/ Unclear)** | 1. have you given your child formula in the last 24 hours?  2. have you breastfed your child in the past 24 hours?  3. Have you fed your child fresh milk such as cow's milk, goat's milk, or yogurt in the past 24 hours?  4. Have you given your child sugar water, juice, tea or drinks in the last 24 hours? |
| **Demographic characteristics** | 1. Your age  2. Your marital status  3. Your occupation prior to production  4. Months of age of the newborn  5. Working status  6. Education  7. Residential structure  8. History of smoking  9. History of drinking  10. Mode of delivery  11. Parity  12. Living place  13. Family history of diabetes (both parents, grandparents) |

- **Supplementary File 2 Analysis of demographic characteristics between 324 respondents and 885 pregnant women with gestational diabetes mellitus**

|  | Respondents(n=401) | Candidates(n=885) | P |
| --- | --- | --- | --- |
|  | n (%) | n (%) |  |
| Age (Years) |  |  |  |
| ＜25 | 9 (2.78) | 17 (1.92) | 0.7404 |
| ≥25-＜30 | 96(29.63) | 266 (30.05) |  |
| ≥30-35 | 140 (43.21) | 390 (44.06) |  |
| ≥35-＜40 | 70 (21.60) | 196（22.14） |  |
| ≥40 | 9 (2.78) | 16（1.80） |  |
| Marriage |  |  |  |
| Married | 323 (99.69) | 878 (99.20) | 0.6061 |
| Unmarried | 1 (0.31) | 7 (0.80) |  |
| Level of education |  |  |  |
| High school or technical secondary school and below | 56 (17.28) | 154(17.40) | 0.974 |
| Junior college | 86 (26.54) | 246 (27.79) |  |
| Undergraduate | 140 (43.21) | 372（42.03） |  |
| Master or above | 42 (12.96) | 113（12.76） |  |
| Mode of delivery |  |  |  |
| Vaginal delivery | 121 (37.35) | 347 (39.21) | 0.6013 |
| Caesarean section | 203(62.65) | 538 (60.79) |  |
| Living place |  |  |  |
| Urban | 304 (93.83) | 810 (91.76) | 0.2314 |
| Rural | 20 (6.17) | 75 (8.24) |  |
| Family of diabetes |  |  |  |
| Yes | 102 (31.48) | 265（29.94） | 0.6567 |
| No | 222 (68.52) | 620（70.06） |  |
| Drinking history |  |  |  |
| Yes | 35 (10.80) | 105 (15.82) | 0.5274 |
| No | 289 (89.20) | 745 (84.18) |  |
| Smoking history |  |  |  |
| Yes | 10 (3.09) | 33 (3.72) | 0.7197 |
| No | 314 (96.91) | 852 (96.28) |  |

- **Supplementary File 3 The Strengthening the Reporting of Observational Studies in Epidemiology (STROBE) checklist**

|  | | **Item No** | **Recommendation** | **Page  No** |
| --- | --- | --- | --- | --- |
| **Title and abstract** | | 1 | (*a*) Indicate the study’s design with a commonly used term in the title or the abstract | 1-2 |
|  |  |  | (*b*) Provide in the abstract an informative and balanced summary of what was done and what was found | 1-2 |
| **Introduction** | | | | |
| Background/rationale | | 2 | Explain the scientific background and rationale for the investigation being reported | 4 |
| Objectives | | 3 | State specific objectives, including any prespecified hypotheses | 5-6 |
| **Methods** | | | | |
| Study design | | 4 | Present key elements of study design early in the paper | 6-7 |
| Setting | | 5 | Describe the setting, locations, and relevant dates, including periods of recruitment, exposure, follow-up, and data collection | 6 |
| Participants | | 6 | (*a*) *Cohort study*—Give the eligibility criteria, and the sources and methods of selection of participants. Describe methods of follow-up  *Case-control study*—Give the eligibility criteria, and the sources and methods of case ascertainment and control selection. Give the rationale for the choice of cases and controls  *Cross-sectional study*—Give the eligibility criteria, and the sources and methods of selection of participants | 8 |
|  |  |  | (*b*) *Cohort study*—For matched studies, give matching criteria and number of exposed and unexposed  *Case-control study*—For matched studies, give matching criteria and the number of controls per case |  |
| Variables | | 7 | Clearly define all outcomes, exposures, predictors, potential confounders, and effect modifiers. Give diagnostic criteria, if applicable | 6-8 |
| Data sources/ measurement | | 8* | For each variable of interest, give sources of data and details of methods of assessment (measurement). Describe comparability of assessment methods if there is more than one group | *6-8* |
| Bias | | 9 | Describe any efforts to address potential sources of bias | 8 |
| Study size | | 10 | Explain how the study size was arrived at | 8 |
| Quantitative variables | | 11 | Explain how quantitative variables were handled in the analyses. If applicable, describe which groupings were chosen and why | 9 |
| Statistical methods | | 12 | (*a*) Describe all statistical methods, including those used to control for confounding | 9 |
|  |  |  | (*b*) Describe any methods used to examine subgroups and interactions | 9 |
|  |  |  | (*c*) Explain how missing data were addressed | 9 |
|  |  |  | (*d*) *Cohort study*—If applicable, explain how loss to follow-up was addressed  *Case-control study*—If applicable, explain how matching of cases and controls was addressed  *Cross-sectional study*—If applicable, describe analytical methods taking account of sampling strategy |  |
|  |  |  | (*e*) Describe any sensitivity analyses |  |
| **Results** | | | | |
| Participants | 13* | (a) Report numbers of individuals at each stage of study—eg numbers potentially eligible, examined for eligibility, confirmed eligible, included in the study, completing follow-up, and analysed | | 9 |
|  |  | (b) Give reasons for non-participation at each stage | | 9 |
|  |  | (c) Consider use of a flow diagram | |  |
| Descriptive data | 14* | (a) Give characteristics of study participants (eg demographic, clinical, social) and information on exposures and potential confounders | | 9-10 |
|  |  | (b) Indicate number of participants with missing data for each variable of interest | | 9-10 |
|  |  | (c) *Cohort study*—Summarise follow-up time (eg, average and total amount) | |  |
| Outcome data | 15* | *Cohort study*—Report numbers of outcome events or summary measures over time | |  |
|  |  | *Case-control study—*Report numbers in each exposure category, or summary measures of exposure | |  |
|  |  | *Cross-sectional study—*Report numbers of outcome events or summary measures | | *9-10* |
| Main results | 16 | (*a*) Give unadjusted estimates and, if applicable, confounder-adjusted estimates and their precision (eg, 95% confidence interval). Make clear which confounders were adjusted for and why they were included | | 9-11 |
|  |  | (*b*) Report category boundaries when continuous variables were categorized | | 9-11 |
|  |  | (*c*) If relevant, consider translating estimates of relative risk into absolute risk for a meaningful time period | |  |
| Other analyses | 17 | Report other analyses done—eg analyses of subgroups and interactions, and sensitivity analyses | |  |
| **Discussion** | | | | |
| Key results | 18 | Summarise key results with reference to study objectives | | 12-15 |
| Limitations | 19 | Discuss limitations of the study, taking into account sources of potential bias or imprecision. Discuss both direction and magnitude of any potential bias | | 16 |
| Interpretation | 20 | Give a cautious overall interpretation of results considering objectives, limitations, multiplicity of analyses, results from similar studies, and other relevant evidence | | 13-15 |
| Generalisability | 21 | Discuss the generalisability (external validity) of the study results | | 16 |
| **Other information** | | | | |
| Funding | 22 | Give the source of funding and the role of the funders for the present study and, if applicable, for the original study on which the present article is based | |  |

- **Supplementary File 4 Respondents’ determinants of breastfeeding based on HBM**

**Table S4-1 perceived susceptibility**

| **Items** | **Mean** | **SD** |
| --- | --- | --- |
| 1. How likely do you think you are to develop chronic diabetes in the future? | 2.69 | 0.67 |
| 2. How likely do you think you are to become obese in the future? | 2.71 | 0.73 |
| 3. How likely do you think it is that your child will be obese as an adult? | 2.28 | 0.64 |
| 4. How likely do you think it is that your child will develop chronic diabetes in the future? | 2.45 | 0.65 |
| Overall scores | 2.53 | 0.52 |

**Table S4-2 perceived severity**

| **Items** | **Mean** | **SD** |
| --- | --- | --- |
| 1. If you develop chronic diabetes in the future, do you think its consequences will be serious? | 3.12 | 0.64 |
| 2. If you develop chronic diabetes in the future, how likely do you think you are to have serious adverse consequences of your diabetes (diabetic foot, cataracts, etc.)? | 2.87 | 0.71 |
| 3. How likely are you to experience severe financial burden if you develop chronic diabetes in the future? | 2.93 | 0.73 |
| 4. If your child develops chronic diabetes in the future, do you think the consequences will be serious? | 3.24 | 0.72 |
| 5. If your child develops chronic diabetes in the future, how likely do you think it is that your child will have serious adverse consequences of diabetes (diabetic foot, cataract, etc.)? | 2.77 | 0.76 |
| 6. If your child develops chronic diabetes in the future, how likely do you think it is that your child will have a serious financial burden from diabetes? | 2.84 | 0.80 |
| Overall scores | 2.96 | 0.57 |

**Table S4-3 perceived barrier**

| **Items** | **Mean** | **SD** |
| --- | --- | --- |
| 1. I don't have enough breast milk to allow me to breastfeed exclusively. | 2.39 | 0.94 |
| 2. Breastfeeding affects my body image. | 2.21 | 0.74 |
| 3. I don't have enough time and energy to breastfeed. | 2.08 | 0.78 |
| 4. breast swelling would make me consider giving up breastfeeding. | 1.81 | 0.65 |
| 5. Breastfeeding can make jaundice last longer in babies. | 2.10 | 0.65 |
| 6. it is difficult for me to breastfeed. | 2.10 | 0.74 |
| 7. Breastfeeding interferes with my daily life and rest. | 2.60 | 0.76 |
| 8. I think formula can give babies exactly the same nutrition. | 2.32 | 0.76 |
| Overall scores | 2.20 | 0.47 |

**Table S4-4 perceived benefit**

| **Items** | **Mean** | **SD** |
| --- | --- | --- |
| 1. Breastfeeding is economical. | 3.25 | 0.67 |
| 2. breastfeeding is convenient. | 3.21 | 0.70 |
| 3. breast milk strengthens the child's resistance. | 3.56 | 0.56 |
| 4. breastmilk is rich in nutrients. | 3.54 | 0.54 |
| 5. breast milk is natural and safe. | 3.59 | 0.54 |
| 6. breastfeeding can increase parent-child bonding. | 3.67 | 0.49 |
| 7. Breastfeeding helps me recover faster from childbirth. | 3.32 | 0.73 |
| 8. Breastfeeding reduces the likelihood of my developing diabetes in the future | 3.01 | 0.76 |
| 9. Breastfeeding can reduce the likelihood of my child developing diabetes in the future | 3.01 | 0.76 |
| Overall scores | 3.35 | 0.46 |

**Table S4-5 Social support**

| **Items** | **Mean** | **SD** |
| --- | --- | --- |
| 1. During the feeding of my child, someone was always there for me when I needed help. | 3.56 | 0.99 |
| 2. I always have someone around me to share my joys and sorrows in feeding my children. | 3.65 | 0.96 |
| 3. My family was always trying to help me in the feeding of my child. | 3.74 | 0.97 |
| 4. I received the emotional help and support I needed from my family during the feeding of my child. | 3.78 | 0.97 |
| 5. I have people around me who make me feel really comfortable during the feeding of my child. | 3.71 | 0.98 |
| 6. My friends always try to help me in the feeding process of my children. | 3.74 | 0.86 |
| 7. I can rely on my friends if I encounter difficulties in feeding my child. | 3.55 | 0.98 |
| 8. I can talk to my family about the difficulties I face during the feeding of my child. | 3.81 | 0.93 |
| 9. I have friends who can share my joys and sorrows in feeding my child. | 3.89 | 0.89 |
| 10. having people in my life who care about my feelings during the feeding of my child. | 3.94 | 0.86 |
| 11. my family is willing to help me make decisions when it comes to feeding my child. | 3.59 | 0.97 |
| 12. I can discuss the difficulties I face in feeding my child with my friend. | 3.94 | 0.86 |
| Overall scores | 3.74 | 0.74 |

**Table S4-6 Self-efficacy**

| **Items** | **Mean** | **SD** |
| --- | --- | --- |
| 1. I can always be sure that my baby is getting enough breast milk. | 3.25 | 1.19 |
| 2. I have always managed to cope successfully with the challenges of breastfeeding. | 3.33 | 1.14 |
| 3. I was always able to breastfeed exclusively without formula. | 3.14 | 1.49 |
| 4. I always make sure my baby can hold the nipple well during feeding. | 3.77 | 1.12 |
| 5. I always manage breastfeeding situations in a way that I am satisfied with. | 3.37 | 1.16 |
| 6. I was always able to finish breastfeeding even when my child cried. | 3.48 | 1.19 |
| 7. I was always able to breastfeed willingly from the beginning to the end. | 3.88 | 1.24 |
| 8. I am always comfortable breastfeeding even when my family is around. | 3.23 | 1.28 |
| 9. I am always satisfied with my breastfeeding experience. | 3.26 | 1.37 |
| 10. I have always been able to handle the fact that breastfeeding is time consuming. | 3.52 | 1.23 |
| 11. I can always get my child to empty one breast before sucking the other. | 3.26 | 1.22 |
| 12. I can always use breast milk every time I feed my child. | 3.46 | 1.37 |
| 13. I always find ways to keep up with my child's feeding needs. | 3.61 | 1.20 |
| 14. I always know when my child is finished breastfeeding. | 3.43 | 1.18 |
| Overall scores | 3.43 | 0.97 |

- **Supplementary File 5 Results of covariates on the breastfeeding behavior based on HBM**

| Factor | Covariates effects on each other | β | SE | P |
| --- | --- | --- | --- | --- |
| Perceived susceptibility | Genetic history of diabetes | 0.223 | 0.109 | <0.001 |
| Perceived severity | Age | 0.418 | 0.249 | 0.015 |
|  | Drinking history | 0.174 | 0.143 | 0.010 |
|  | Education | 0.231 | 0.127 | 0.015 |
|  | Occupation | -0.168 | 0.159 | 0.025 |
| Perceived benefits | Education | 0.173 | 0.103 | 0.046 |
|  | Parity | 0.208 | 0.069 | 0.002 |
|  | Genetic history of diabetes | -0.132 | 0.075 | 0.046 |
|  | Drinking history | 0.135 |  |  |
| Perceived barriers | Working status | 0.157 | 0.110 | 0.017 |
|  | Genetic history of diabetes | 0.130 | 0.087 | 0.039 |
| Self- efficiency | Parity | 0.207 | 0.091 | <0.001 |
|  | Occupation | 0.148 | 0.137 | 0.016 |
| Social support | Education | 0.215 | 0.160 | 0.010 |
| Breastfeeding behavior | Education | 0.213 | 0.219 | 0.030 |
|  | Age of neonatal | -0.252 | 0.164 | 0.001 |
